# Effect of Exercise Training on Enos Expression, NO Production and Oxygen Metabolism in Human Placenta

**DOI:** 10.1371/journal.pone.0080225

**Published:** 2013-11-14

**Authors:** Robinson Ramírez-Vélez, Juanita Bustamante, Analia Czerniczyniec, Ana C. Aguilar de Plata, Silvia Lores-Arnaiz

**Affiliations:** 1 Facultad de Cultura Física, Deporte y Recreación, Universidad Santo Tomás, Bogotá, D.C, Colombia; 2 Departamento de Ciencias Fisiológicas, Facultad de Salud, Universidad del Valle, Cali, Colombia; 3 Instituto de Bioquímica y Medicina Molecular (UBA-CONICET), Facultad de Farmacia y Bioquímica, Universidad de Buenos Aires, Buenos Aires, Argentina; Temple University, United States of America

## Abstract

**Objective:**

To determine the effects of combined aerobic and resistance exercise training during the second half of pregnancy on endothelial NOS expression (eNOS), nitric oxide (NO) production and oxygen metabolism in human placenta.

**Methods:**

The study included 20 nulliparous in gestational week 16–20, attending prenatal care at three tertiary hospitals in Colombia who were randomly assigned into one of two groups: The exercise group (n = 10) took part in an exercise session three times a week for 12 weeks which consisted of: aerobic exercise at an intensity of 55–75% of their maximum heart rate for 60 min and 25 mins. Resistance exercise included 5 exercise groups circuit training (50 repetitions of each) using barbells (1–3 kg/exercise) and low-to-medium resistance bands. The control group (n = 10) undertook their usual physical activity. Mitochondrial and cytosol fractions were isolated from human placental tissue by differential centrifugation. A spectrophotometric assay was used to measure NO production in cytosolic samples from placental tissue and Western Blot technique to determine eNOS expression. Mitochondrial superoxide levels and hydrogen peroxide were measured to determine oxygen metabolism.

**Results:**

Combined aerobic and resistance exercise training during pregnancy leads to a 2-fold increase in eNOS expression and 4-fold increase in NO production in placental cytosol (p = 0.05). Mitochondrial superoxide levels and hydrogen peroxide production rate were decreased by 8% and 37% respectively in the placental mitochondria of exercising women (p = 0.05).

**Conclusion:**

Regular exercise training during the second half of pregnancy increases eNOS expression and NO production and decreases reactive oxygen species generation in human placenta. Collectively, these data demonstrate that chronic exercise increases eNOS/NO production, presumably by increasing endothelial shear stress. This adaptation may contribute to the beneficial effects of exercise on the vascular and antioxidant system and in turn reduce the risk of preeclampsia, diabetes or hypertension during pregnancy.

## Introduction

Pregnancy is a physiological state characterized by hemodynamic changes, such as an increase in heart rate at rest, increased cardiac output and diminished peripheral vascular resistance [1]. These adaptations appear to be mediated to a great extent by an increased endothelium-dependent vasodilation. The endothelial release of nitric oxide (NO) has been proposed as the main factor responsible for the decreased systemic vascular resistance observed during pregnancy [1]. In addition, a decreased production or an increased inactivation of NO has been linked to the endothelial dysfunction that characterizes pre-eclampsia, gestational diabetes and hypertension [2]. Moreover, while pregnancy is referred to as “a state of oxidative stress” [3], certain pathological conditions, such as pre-eclampsia further increase placental oxidative stress and may result in early pregnancy loss, intrauterine growth restriction (IUGR) and impaired placentation [4]. Placental mitochondria are one of the a major source of oxidative stress in pre-eclampsia [3], [4]

In recent years, evidence has accumulated to support the popular belief that physical activity is associated with better psychological health during pregnancy [5], [6]. The American College of Obstetricians and Gynecologists (ACOG) [7] recommend regular exercise for pregnant women, including those who are sedentary, for its overall benefits on physical and psychological health. Physical activity during pregnancy appears to be beneficial to the maternal-foetal unit [8] and may prevent the occurrence of maternal disorders, such as preeclampsia [9], hypertension [10] and gestational diabetes [11]. Other studies have demonstrated that either beginning or continuing regular weight-bearing exercise throughout pregnancy improves placental growth [12], [13]. Bergmann et al. [14] reported that regular weight-bearing forms of exercise influence placental growth and anatomic indices of functional capacity. Our previous work, showing that 12 weeks of exercise enhances endothelium-dependent brachial artery dilation in pregnant women suggested that exercise-induced increases both blood flow and eNOS expression [15]. The exact mechanism for this remains unclear, but it is proposed that the exercise-induced intermittent fluctuations in substrate and oxygen delivery produce a recurrent stimulus which evokes an overall growth response [8]–[10].

It is currently unknown, however if exercise produces these effects in human placenta. The aim of this study was to determine the effects of exercise training during the second half of pregnancy effect on endothelial NOS expression (eNOS), nitric oxide (NO) production and oxygen metabolism in human placenta.

## Methods

### Subjects

Twenty nulliparous women with a gestational age ranging 16–20 weeks of healthy pregnancy and a live fetus at the routine ultrasound scan were included in the study. Participants were originally deemed eligible for this investigation if they met all of the following criteria in the first trimester of pregnancy: *i)* gravida with singleton and uncomplicated gestation; *ii)* not at high risk for preterm delivery (p.e: alcohol use, drug use, history of smokers, placenta previa, incompetent cervix, polyhydramnios, oligohydramnios, or miscarriage in the last twelve months); *iii)* 18–25 years of age; *iv)* being sedentary before gestation (exercising <20 min on <3 days/week); *v)* being under medical follow-up throughout the entire pregnancy period (and planning to give birth) in the same obstetrics hospital department (Red de Salud Ladera, Cali-Colombia); and *vi)* having no absolute or relative contraindication to exercise participation during pregnancy (such as, among others, haemodynamically significant heart disease, restrictive lung disease, pregnancy induced hypertension, severe anaemia, maternal cardiac arrhythmia, chronic bronchitis, type I diabetes or extreme morbid obesity (body mass index ≥40 kg/m^2^)). Maternal and fetal characteristics are presented in (Table 1.)

**Table 1:** Characteristics of the population: maternal age, BMI, gestational age at birth, fetal and placental weight and fetal gender in pregnancies.

Characteristics	Control	Exercised
**Maternal age (yr)**	19.5±3.4	19.2±2.6
**Body mass index (BMI)**	24.1±4.5	22.9±3.8
**Gestational age at birth (yr)**	39.8±2.0	38.7±1.0
**Fetal weight (g)**	3013.2±493.8	3133.3±406.5
**Placental weight (g)**	430.4±28.9	390.0±20.4
**Fetal gender (female/male)**	6/4	5/5

Data reported are mean ± SD.

The Ethics Committees of Universidad del Valle and Red de Salud Ladera (Resolution-017/08-UV and SCAH/0408-A/08) approved the trial. All participants provided written informed consent before entering the study. Participants and their legal representatives received information sheets and all provided written informed consent and approval to take part in the study. The protocol was in accordance with the latest revision of the Declaration of Helsinki.

### Experimental design

This study involved a subset of subjects enrolled in a randomized controlled trial to evaluate the effect of regular exercise on maternal endothelium-dependent vasodilatation (A detailed account of the methods used in this study has been published elsewhere [15], from March 2010 to January 2011.

### Interventions and training program

The pregnant women who were assigned to the exercise group participated in a 60 min supervised aerobic and resistance exercise session, three times a week [15], [16]. An average of 32 training sessions per subject was planned for each participant, in consideration that delivery could occur before 40 weeks' gestation. All women had their heart rate monitored during the training sessions to ensure that the exercise was of a moderate-to vigorous intensity. A physiotherapist and a physical educator were present during the exercise sessions. Each session included 30-min of aerobic circuit training accompanied by an audio recording of music and instructions which guided the participants to exercise at each station for approximately 1 min and then move to the next station in three circuits of 10 stations. Participants wore a heart rate monitor (Polar Pacer, USA) in every exercise session which were checked at rest after 15 and 30 mins to ensure that they exercised in the prescribed target heart rate zone. Each session was preceded and followed by a gradual warm-up and cool down period which were both of 10 min duration and consisted of walking and light, static stretching (avoiding muscle pain) of major muscle groups (upper and lower limbs, neck and trunk muscles). The cool down period also included relaxation and stretching exercises.

Resistance exercises were performed through the full range of motion normally associated with correct technique for each exercise and engaged the major muscle groups (abdominal, dorsal, shoulder, upper and lower limb muscles). They included 5 exercise groups circuit training (50 repetitions of each) using barbells (1–3 kg/exercise) or low-to-medium resistance bands (Therabands). Each type of exercise on the back was performed for 2 min. As a general rule and to reduce potential risks, we excluded activities that promoted that Valsalva maneuver, ballistic or plyometric exercises and positions of extreme muscular tension.

The control group received no intervention; that is, participants neither attended the exercise classes nor took part in an exercise program at home. Both groups continued with their normal prenatal care and physical activity. Each woman met with the study dietician for nutrition assessment and counselling, and an individualized nutrition intervention plan was developed from the baseline food intake assessment, participant preferences, and the meal plan [16], [17]. On a weekly basis, each participant all participants received a light breakfast/meal 45 min before the exercise session which typically consisted of a meal designed to promote weight and recommended during pregnancy (approximately (400 kcal) [carbohydrate to 40–55%, fat 30% and 20–30% to protein of total energy) [17]. The food records were analyzed for nutritional content and caloric intake using the ESHA Food Processor SQL (version 9.8; Canadian Nutrient File database). The participants received support for public transport to attend the exercise classes (US10 per session).

### Tissue preparation and sampling

Placentas were collected within 15 min of delivery. The maternal decidua was removed and the central portion between the maternal and fetal surfaces used in the preparation. Maternal villous tissue (100 g) was chopped into small pieces, washed with NaCl 0.9 per cent to remove blood and filtered through gauze.

### Isolation of human placental mitochondria

Human placental mitochondria were obtained with the modified protocol of differential centrifugation, previously described by Martinez et al. [18], [19]. Briefly, several placental cotyledons were removed from the maternal side of the placenta and placed immediately into an ice-cold medium containing MSHE buffer (210 mM mannitol, 70 mM sucrose, 5 mM Hepes, 1 mM EDTA, pH 7.4). All steps were carried out at 4°C. Soft villous tissue (4–7 g) was freed from connective tissue and minced into small pieces with scissors. The tissue was washed with the same solution and then filtered three times through a thin surgical gauze layer. The tissue was re-suspended in the same medium and homogenized with a Potter-Elvehjem homogenizer (7 up-and-down strokes). The homogenate was centrifuged at 1,500 g for 10 min in a refrigerated centrifuge. The supernatant was recovered and centrifuged at 4,000 g for 15 min to pellet large mitochondria (predominantly cytotrophoblast mitochondria) was resuspended in a minimal volume of respiratory medium containing MSH buffer (210 mM mannitol, 70 mM sucrose, 5 mM Hepes, pH 7.4) adjusted to pH 7.4. A part of the isolated mitochondrial fraction was frozen at −80°C until the enzyme assays were performed. This method has previously been shown to yield mitochondria free from contaminants from other parts of the cell. This method yields a total mitochondrial protein content estimated between 1–2 mg/g of wet placental tissue.

### Endothelial NOS expression

The frozen placental tissue were thawed and minced into small pieces and homogenized in cell lysis buffer containing 100 mM Tris, 50 mM NaCl, 10 mM EDTA, 1% TritonX-100. Insoluble placenta tissues were removed by centrifugation at 3.000 g, 4°C, for 10 min. Samples were loaded and subjected to SDS-PAGE in 7.5% polyacrylamide gels. After electrophoresis, proteins were electro-transferred to nitrocellulose membrane (Amersham Biosciences; Piscataway, NJ). Equal loading of samples (80 µg) and even transfer efficiency were monitored with the use of 0.5% Ponceau S staining of the blot membrane. The blot membrane was then incubated in a blocking buffer (5% nonfat dry milk, 10 mM Tris-HCl, pH 7.6, 150 mM NaCl, and 0.1% Tween 20) for 2 h at room temperature and then probed with a rabbit polyclonal antibody against the endothelial isoform of NOS (amino terminus, H-299, Santa Cruz Biotechnology, Santa Cruz, CA, USA: dilution 1∶500) at room temperature. The nitrocellulose membrane was subsequently incubated with a secondary goat anti-rabbit antibody conjugated with horseradish peroxidase (HRP) (dilution 1∶1000), and revealed by chemiluminescence with ECL reagent (Amersham Biosciences; Piscataway, NJ). Densitometric analysis of the eNOS bands was performed using the NIH Image 1.54 software. β-actin expression levels were used to normalize the results [20].

### NOS activity

Nitric oxide production was measured in cytosolic fractions using a spectrophotometric method by following the oxidation of oxyhemoglobin to methemoglobin at 37°C, in a reaction medium containing 50 mM phosphate buffer (pH 5.8 for mitochondrial preparations and pH 7.4 for the cytosolic fractions), 1 mM CaCl_2_, 50 µM L-arginine, 100 µM NADPH, 10 µM dithiothreitol (DTT), 4 µM Cu-Zn superoxide dismutase (SOD) (to avoid interference by O_2_^−^), 0.1 µM catalase (to avoid oxyhemoglobin oxidation by H_2_O_2_), 0.5–1.0 mg submitochondrial protein/ml and 25 µM oxyhemoglobin (expressed per heme group). Kinetics were followed at 577–591 nm (ε = 11.2 mM^−1^ cm^−1^) in a double-beam double wave length spectrophotometer (Beckman-Coulter Series DU) [21].

### Oxygen metabolism

Superoxide anion (O_2_^−^) was detected using the fluorogenic dye MitoSOX (C_43_H_34_N_3_IP) a membrane permeant and rapidly targeted mitochondrial O_2_^−^ indicator for living cells, with excitation/emission maxim of 510/580 nm. Based on the ability of the positively charged TPP^+^ moiety of MitoSox Red to accumulate in the mitochondrial matrix, and also due to its ability to intercalate into mtDNA, a sufficient amount of this probe is retained in the matrix. Both MitoSox Red free and DNA-intercalated forms are sensitive to O_2_^−^. We developed a special protocol by flow cytometry in order to detect superoxide anion not consumed by the MnSOD in isolated mitochondria. Isolated mitochondria from placental tissue were loaded with 2.5 µM MitoSOX, during 20 min at 37°C, in the same MSH buffer supplemented with malate plus glutamate. Antimycin A (0.5 µM), an inhibitor of the ubiquinone–cytochrome c reductase, was used as a positive control. Auto fluorescence was evaluated in samples without probe. Hydrogen peroxide (H_2_O_2_) generation was determined in intact isolated human placental mitochondria by the scopoletin-HRP method following the decrease in fluorescence intensity at 365–450 nm (λ exc-λ em) at 37°C [21]. The reaction medium consisted of 0.23 M mannitol, 0.07 M sucrose, 20 mM Tris-HCl (pH 7.4), 0.8 µM HRP, 1 µM scopoletin, 0.3 µM SOD to ensure that all O_2_^−^ was converted to H_2_O_2_; 6 mM succinate plus glutamate were used as substrates. Calibration was made using H_2_O_2_ (0.05–0.35 µM) as standard to express the fluorescence changes as nmol H_2_O_2_/min.mg protein. H_2_O_2_ production was highly sensitive to catalase addition (3.500 U/ml).

### Maternal and Conceptus morphometrics

Study investigators were informed when any study participant was admitted for delivery, and were present to monitor labor and delivery and to collect data. Gestational age at the time of delivery (in weeks and days) was recorded from hospital perinatal records. Anthropometric measurements of newborns: birth weight and placental weight (SECA scale ±10 g), were taken one hour after delivery by standard methods [13], [14]. Placental efficiency was defined as the fetal weight/placental weight, as described previously [13], [14]

### Protein assays

Determination of mitochondrial protein was determined by the Lowry assay [22] using bovine serum albumin as the standard (Bio-Rad Protein Assay). All experiments were performed in triplicate.

### Statistical analysis

All the data were subjected to statistical analyses using the SAS program. Maternal and fetal characteristics presented were expressed as means ± SE. Values in figures are the mean ± SEM. Statistical comparisons of superoxide anion level and NO production by groups were performed with two-way ANOVA. Intra-group and patient comparisons of L-NAME *or* Antimycin are tested with Wilcoxon test. The eNOS activity, H_2_O production, placental weight and placental efficiency were compared by Wilcoxon test. A difference was considered to be statistically significant when p<0.05.

## Results

### Compliance with the trial method

From enrollment to at least 28–32 wk gestation, the mean participation of the active subjects was 28.9 out of 36 (SD 3.2) sessions over the 12 weeks. No participant experienced adverse events during or after the exercise.

### eNOS expression and NO production

**Figure 1:**
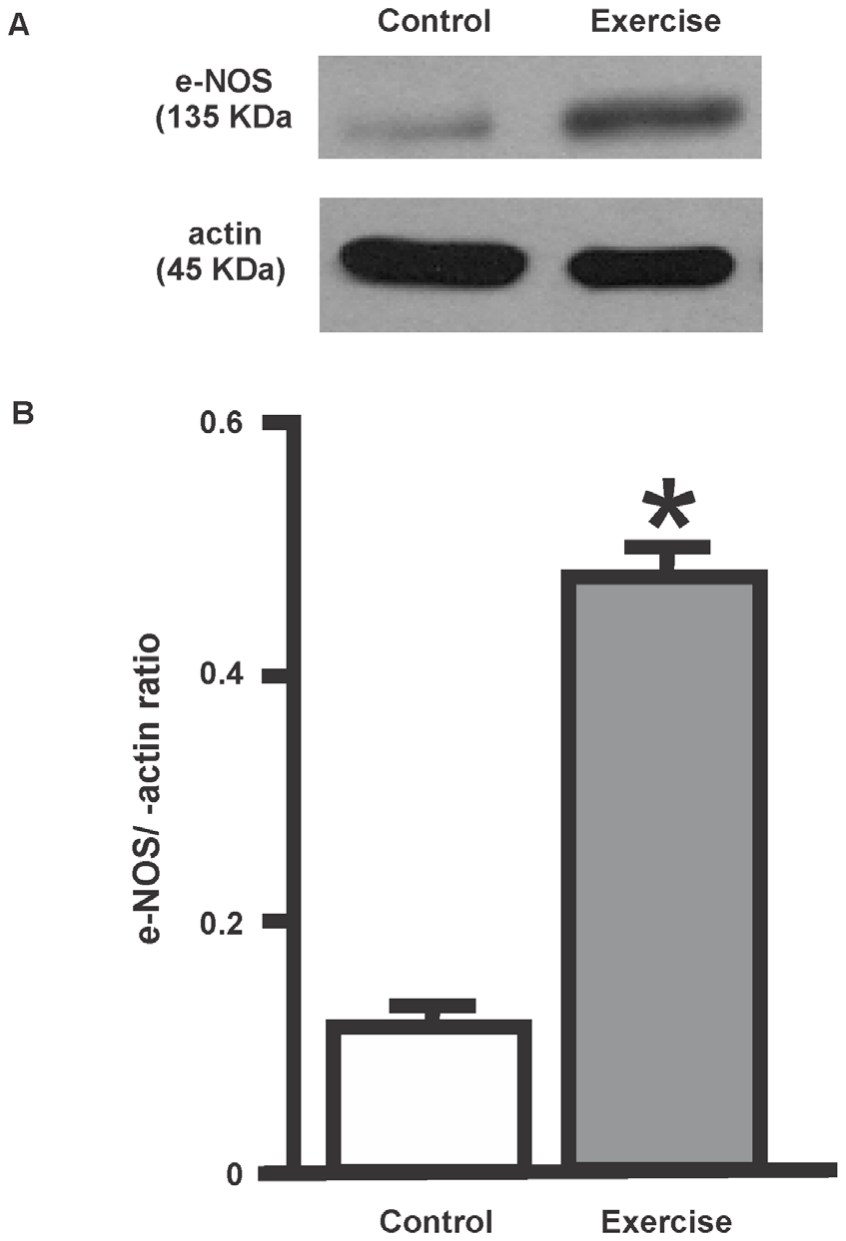
Effects of physical exercise on eNOS protein expression. **A**. Typical examples of Western blots for placental cytosolic fractions for each experimental group. Each blot was normalized to expression of β-actin from the same gel. **B**. Bars represent cytosolic eNOS/β-actin ratios ± SEM obtained after densitometric analysis. (*p<0.05, as compared with control value). The results shown are representative of three independent studies.

ure 1 shows that in pregnant women 12 weeks of exercise training resulted in a 4-fold increase in eNOS/β-actin expression in cytosolic samples (p<0.05). A 2-fold increase in NO production was also observed in their cytosolic samples (p<0.05) (Fig 2).

**Figure 2:**
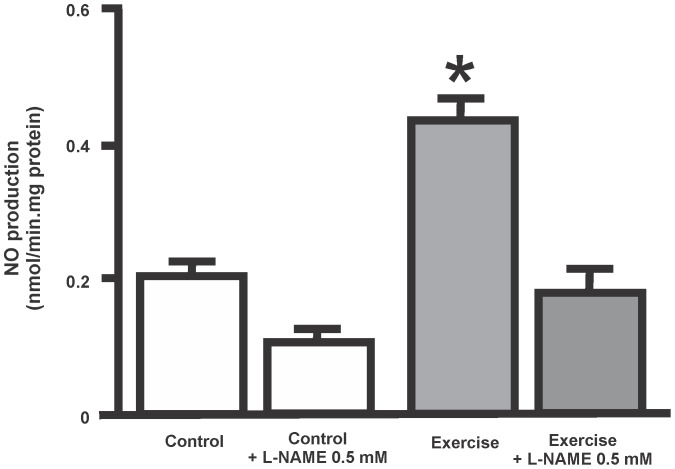
Effects of physical exercise on NO production in cytosolic samples from placental tissue. The specificity of the assay was evaluated using 0.5-NAME. Data are expressed as mean ± SEM. (*p<0.05, as compared with control value).

### Oxygen metabolism

**Figure 3:**
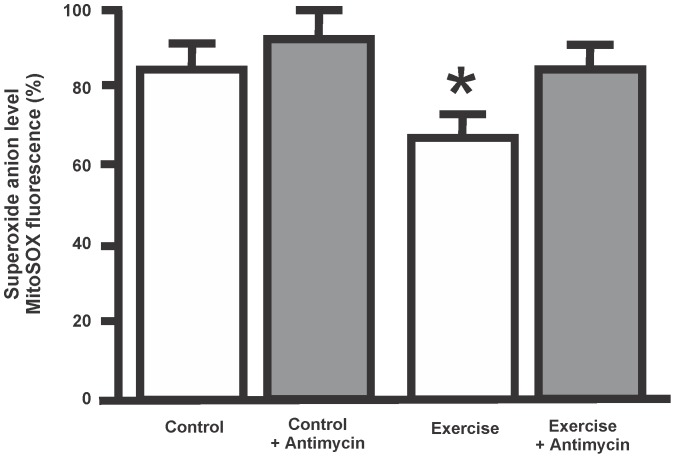
Determination of superoxide anion level. Mitochondrial samples were loaded with the superoxide anion sensor MitoSox during 20°C. Antimycin A (0.5 µM) was used as a positive control. Bar graph quantification of Mitosox fluorescence expressed as percentage values of human placental mitochondria from control and exercised women. Antimycin effect is also shown for both samples. Data are expressed as mean ± SEM, from three independent experiments. (*p<0.05, as compared with control value).

Within the mitochondria, the electron transport chain is the main source of reactive oxygen species, O_2_^−^ being the primary molecule generated by the mitochondrial respiratory chain. The mitochondria were loaded with MitoSOX, a fluorescence probe that typically increases its emission in the presence of an excessive generation of O_2_^−^ escaping from the action of the MnSOD. Quantification of the superoxide anion levels as percentage values of MitoSox r. f. i is shown in Fig. 3. Mitosox fluorescence intensity was 8% lower in exercised mitochondria than in control samples. As expected, both mitochondrial samples exposed to antimycin showed higher FL-2 fluorescence values as compared with basal levels.

**Figure 4:**
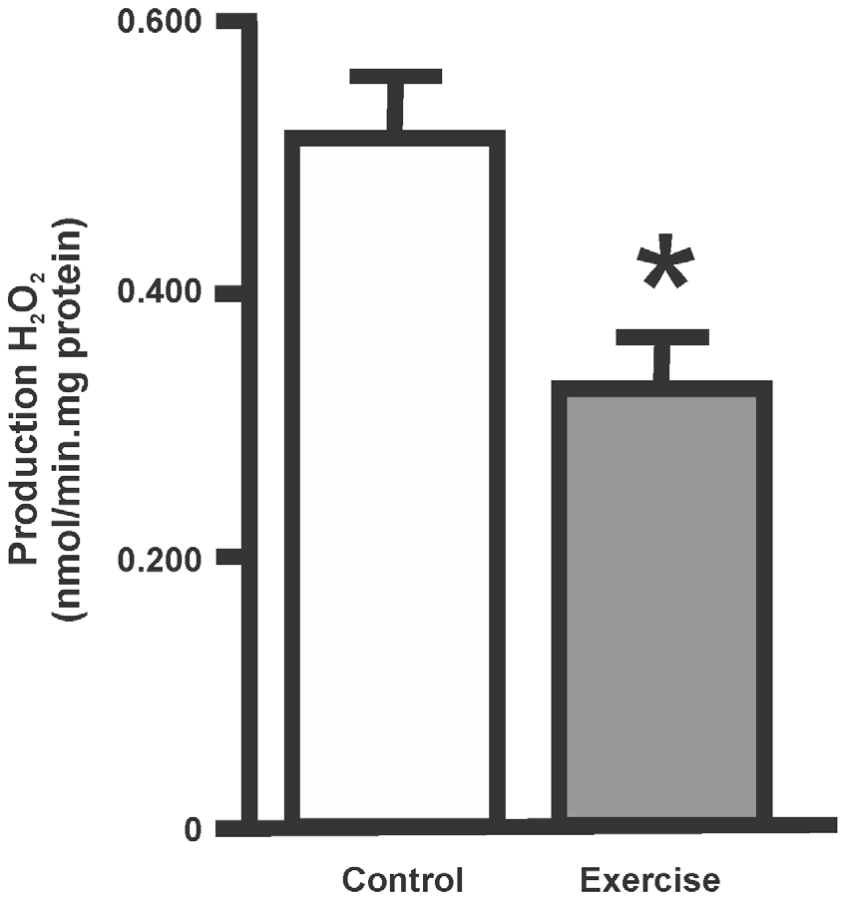
Bar scheme of H_2_O_2_ production rate of human placental mitochondria from exercised or control subjects. Data are expressed as mean ± SEM of 4–6 mitochondrial samples for each condition. (*p<0.05, as compared with control value).

H_2_O_2_ production was measured in intact isolated human placental mitochondria from control and exercised women, using succinate-glutamate as mitochondrial substrates. Hydrogen peroxide rates obtained from human placenta mitochondria were within the range of H_2_O_2_ production rates previously observed in our laboratory for other tissues e.g. rodent heart, liver and brain [19], [23]. Mitochondrial H_2_O_2_ production rate was approximately 0.51±0.06 nmol/min.mg protein for placental mitochondria isolated from the control women. A 37% decrease of H_2_O_2_ production rate was observed in the placental mitochondria of exercised women, compared with control subjects (p<0.05) (Fig. 4).

### Maternal and Conceptus morphometrics

**Figure 5:**
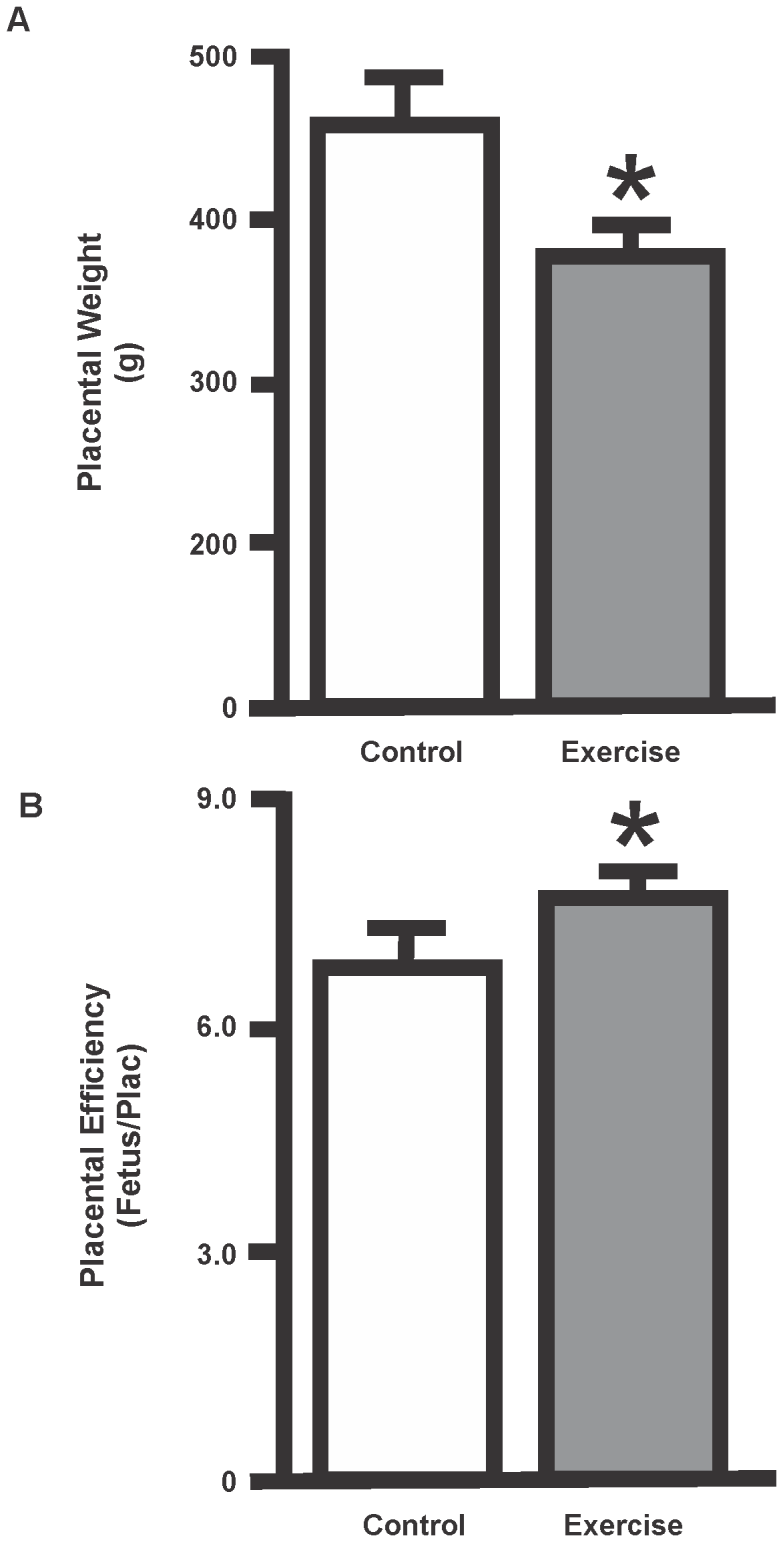
Effects of exercise on placental weight and placental efficiency. **A.** Placental weight was decreased in the exercise trained compared to the non-exercised. **B.** The placental efficiency was increased in the exercise group compared to the control group. Data are expressed as mean ± SEM. (*p<0.05, as compared with control value).

Fetal weight (3013.2±493.8 vs. 3133.3±406.5 g) was not altered in the exercise trained compared to the non-exercise group. In contrast, placental (Figure 5A; p<0.05) weight decreased in the exercise trained compared to the non-exercised. Furthermore, placental efficiency was increased (Figure 5B; p<0.05) in the exercise trained. There was no difference in the number of cotyledons (12.5±0.5 vs. 12±1.0) between the exercise and non-exercise groups.

## Discussion

The present study reveals several interesting and novel findings regarding maternal and placental adaptations to chronic exercise training during pregnancy. Exercise training during pregnancy led to a 2-fold increase in eNOS expression and a 4-fold increase in NO production in placental cytosol. Exercised women also showed 6% decreases in mitochondrial O_2−_ levels and 26% decreases in H_2_O_2_ production rate in placental mitochondria. NO generated by NOS has been demonstrated to contribute to the regulation of vascular tone by counteracting the actions of vasoconstrictors [24]. NOS in the placental villous vasculature also corresponds to the type III calcium-calmodulin-dependent endothelial isoform, and no such NOS was found in the cytotrophoblast layer or in small fetal blood capillaries of term placenta [25]. According to the present findings, a combined resistance and aerobic exercise training program leads to an increased eNOS expression and NO production in human placental tissue. Potentially, the underlying mechanisms may relate to an exercise induced increase in shear stress on the endothelial monolayer, in turn enhancing the activity of enzymes such as eNOS and extracellular superoxide dismutase and shifting the reactive oxygen balance toward the beneficial NO [26]. However, other vasoactive substances such as prostacyclin, or endothelium-derived hyperpolarizing factor (EDHF) could also be involved [1], [2]. It has been reported that exercise produces a state of hyperemia and an increase of blood flow and shear stress with a consequent increase in the production and bioactivity of NO [15], [16]. However, parallel or subsequent events are less clear, and whether the improvement in endothelium function induced by exercise during pregnancy observed in the present study has a beneficial effect in the prevention of these disorders (i.e., reduced risk of preeclampsia, diabetes or hypertension), is yet to be determined.

The placenta generates reactive oxygen species which may contribute to the oxidative stress seen even in normal pregnancy, but this is increased in pregnancies complicated by pre-eclampsia, IUGR and gestational diabetes where elevated oxidative and nitrative stress have been clearly documented [27]. Nitrative stress is the covalent modification of proteins and DNA by H_2_O_2_ formed by the interaction of O_2−_ and NO [28]. In the present study, reactive oxygen species O_2−_ and H_2_O_2_ were significantly diminished in human placental mitochondria from exercised women as compared with control subjects, suggesting that chronic exercise training results in a lower level of oxidative stress. Studies by Bo et al. [29] showed that during prolonged exercise, UCP2 mRNA expression and activity in rat heart can be upregulated, thereby reducing cross-membrane Δψ and reactive oxygen species production. One of the important effects of increased UCP2 expression is to decrease the generation of oxygen free radicals in the mitochondria. These results were in agreement with studies by Venditti et al. [30] who found that succinate-supported H_2_O_2_ release was higher in skeletal muscle from trained rats in both State 4 and State 3, while training did not affect mitochondrial oxygen consumption with both complex-I- and complex II-linked substrates.

We also report that exercise training during pregnancy was associated with a decreased placental weight, but an increased placental efficiency (fetal weight: placental weight). Previous studies have reported a wide range of effects of chronic exercise before and during pregnancy on conceptus weight in both humans [13], [14] and rats [31]. The underlying differences between these observations remain unclear but the wide variation in the amounts and types of exercise are likely contributing factors. Further studies are needed to determine if other indices of placenta function such as transport of amino acids and vascular density are improved by exercise training.

More laboratory based studies and clinical trials are needed to confirm and elaborate the effects of aerobic and resistance exercises as the small sample size, warrants caution in the interpretation of the results. Nevertheless, these results may offer a plausible explanation for previous reports showing a decreased incidence of preeclampsia in woman participating in exercise training [32] and are consistent with findings from previous studies in other tissues [33].

As a general conclusion, regular exercise training during the second half of pregnancy increases eNOS expression and NO production and decreases reactive oxygen species generation in the mitochondrial respiratory chain in placental mitochondria. This finding provides a pathophysiologic framework for the elucidation of the positive effects of exercise on placental human and demonstrates the therapeutic potential of exercise training to improve fetal oxygenation and in turn potentially reduce risk of gestational disorders associated with impaired endothelial function [34].
